# *De Novo* Transcriptome Assembly in *Shiraia bambusicola* to Investigate Putative Genes Involved in the Biosynthesis of Hypocrellin A

**DOI:** 10.3390/ijms17030311

**Published:** 2016-02-27

**Authors:** Ning Zhao, Xi Lin, Shan-Shan Qi, Zhi-Mei Luo, Shuang-Lin Chen, Shu-Zhen Yan

**Affiliations:** College of Life Sciences, Nanjing Normal University, Nanjing 210023, China; zn72795@163.com (N.Z.); 18751880150@163.com (X.L.); qi.305@osu.edu (S.-S.Q.); 15836067824@163.com (Z.-M.L.); chenshuanglin@njnu.edu.cn (S.-L.C.)

**Keywords:** *Shiraia bambusicola*, ultraviolet mutagenesis, RNA-Seq, differentially expressed genes

## Abstract

*Shiraia bambusicola* is a species of the monotypic genus *Shiraia* in the phylum Ascomycota. In China, it is known for its pharmacological properties that are used to treat rheumatic arthritis, sciatica, pertussis, tracheitis and so forth. Its major medicinal active metabolite is hypocrellin A, which exhibits excellent antiviral and antitumor properties. However, the genes involved in the hypocrellin A anabolic pathways were still unknown due to the lack of genomic information for this species. To investigate putative genes that are involved in the biosynthesis of hypocrellin A and determine the pathway, we performed transcriptome sequencing for *Shiraia bambusicola* S4201-W and the mutant S4201-D1 for the first time. S4201-W has excellent hypocrellin A production, while the mutant S4201-D1 does not. Then, we obtained 38,056,034 and 39,086,896 clean reads from S4201-W and S4201-D1, respectively. In all, 17,923 unigenes were *de novo* assembled, and the N50 length was 1970 bp. Based on the negative binomial distribution test, 716 unigenes were found to be upregulated, and 188 genes were downregulated in S4201-D1, compared with S4201-W. We have found seven unigenes involved in the biosynthesis of hypocrellin A and proposed a putative hypocrellin A biosynthetic pathway. These data will provide a valuable resource and theoretical basis for future molecular studies of hypocrellin A, help identify the genes involved in the biosynthesis of hypocrellin A and help facilitate functional studies for enhancing hypocrellin A production.

## 1. Introduction

*Shiraia bambusicola* is a species of the monotypic genus *Shiraia* in the phylum Ascomycota. It is known to grow in only some provinces south of the Yangtze River of China and in Japan [1,2]. In China, it has long been used as a traditional medicine for the treatment of rheumatic arthritis, sciatica, pertussis, tracheitis and so on. Its major active metabolic product is hypocrellin (hypocrellin A, hypocrellin B, hypocrellin C, hypocrellin D), which is a perylenequinoid pigment. Hypocrellin A (HA) is the main component in hypocrellin, and it has been found that HA has antiviral and antitumor activities [3,4,5,6]. As a photosensitizer, it can be activated and can generate molecular singlet oxygen (^1^O_2_) and superoxide radicals (O_2_^−^) in the presence of oxygen, which can damage cells [7,8]. HA showed great potential in clinical photodynamic therapy [9]. Although HA has exhibited a variety of pharmacological functions and has been used widely, its anabolic pathway remains unknown.

Perylenequinones, including cercosporin, elsinochrome, phleichrome, cladochrome and hypocrellin, are similar in structure and red in color [10]. The hypocrellin A has been well studied, due to its pharmaceutical potential as anticancer agents through photodynamic tumor therapy. However, cercosporin biosynthesis has been studied long-term and has been used as a model for resistance to ^1^O_2_ and other oxidative stress [11]. Therefore, we can use the working model of cercosporin biosynthetic pathway to study the biosynthesis of HA. In the 1970s, using a substrate-feeding study with acetate and malonate, Okubo *et al.* concluded that cercosporin biosynthesis occurs via a fungal polyketide pathway [12]. Eight co-regulated genes (CTB1–8) were identified in the cluster using a chromosome walking strategy. The CTB1 (Cercosporin Toxin Biosynthesis 1) gene, a polyketide synthase gene, encodes a protein of 2196 amino acids that synthesizes the polyketomethylene backbone of cercosporin [13]. The CTB3 gene includes two putative domains, a flavin adenine dinucleotide (FAD)-dependent monooxygenase domain in the C-terminus and an *O*-methyltransferase domain in the N-terminus [14]. The CTB4 gene is a major facilitator superfamily (MFS) transporter [15]. The CTB5 and CTB7 genes encode a FMN/FAD-dependent oxidoreductases, and CTB6 encodes a NADPH-dependent oxidoreductase [16]. CTB2 encodes an *O*-methyltransferase gene involved in cercosporin biosynthesis. The CTB8 protein has similarity to many fungal transcription factors, which contain zinc finger DNA-binding domains, and CTB8 controls the expression of all of the core cercosporin cluster genes [17]. Chen *et al.* proposed a possible pathway for cercosporin biosynthesis in *Cercospora nicotianae* and paved the way for future research of other perylenequinones. In addition, the first gene putatively identified in the pathway was cercosporin facilitator protein (CFP), which has high homology with MFS drug resistance transporter proteins and was found to be required for exportation of cercosporin from the mycelium of *Cercospora kikuchii* [18]. At present, there is little research in the iterature focused on the molecular genetics of *Shiraia bambusicola*. Chang *et al.* obtained one fungal polyketide synthase gene named *g4 PKS* from *Shiraia* sp. SUPER-H168, and amplified one keto-synthase (PKS) domain fragment of *g4 PKS*. The expression vector pCold II-KS was constructed and expressed in *Escherichia coli* BL21 (DE3) [19]. One type III PKS gene was obtained from *Shiraia* sp. Slf14, which is the key synthase in the product process of the HA [20]. Notwithstanding, the putative genes and the mechanism of the HA biosynthetic pathway remains unclear.

Next-generation sequencing (NGS) technology has recently been widely used in diverse researches to provide a large amount of genetic data of certain species. Compared with time- and energy-consuming traditional methods, RNA-Seq has already been shown to be highly accurate and, with a large dynamic range of expression levels, it requires less RNA to test gene expression of each transcript under different conditions. RNA-Seq technology provides a fast and accurate way to generate large amounts of sequenced transcriptome for these non-model species without reference genome sequence information [21].

To investigate putative genes involved in the biosynthesis of HA and propose a putative biosynthetic pathway, firstly we screened a hypocrellin-producing strain S4201-W, which was isolated from the fruiting bodies of the *Shiraia* *bambusicola* P. Henn. Then we irradiated S4201-W by means of ultraviolet ray, and obtained mutant S4201-D1 that did not produce HA. We performed transcriptome sequencing for them for further analysis. In this study, the Illumina HiSeq^TM^ 2500 platform was used to sequence the transcriptome of *Shiraia bambusicola* to study its molecular genetics and HA biosynthesis. In the future, this study will provide an important resource for gene discovery and increase HA production in *Shiraia bambusicola* through further genetic manipulation.

## 2. Results and Discussion

### 2.1. Transcriptome Sequencing and Assembly

The *S. bambusicola* strains were cultured on potato dextrose agar (PDA, 200 g/L potato extract, 20 g/L glucose, 20 g/L agar, pH 7.0) and potato dextrose broth (PDB, 200 g/L potato extract, 20 g/L glucose, pH 7.0) (shown in Supplementary Figure S1). The concentration of HA was measured by high-performance liquid chromatography (HPLC) with HA standard reagent (Figure S2). As a result, no HA could be detected by HPLC in mutant S4201-D1. According to the standard curve, we know that the yield of HA was about 156.67 mg/L broth in wild type S4201-W. This was prepared for later experiment and analysis. To obtain an overview of the *de novo* transcriptome of *S. bambusicola*, cDNA libraries were constructed and sequenced using the Illumina HiSeq^TM^ 2500 platform, generating 38,230,680 and 39,301,794 raw reads. After removing the adapter sequence and low-quality reads, we obtained 38,056,034 and 39,086,896 clean reads from the S4201-W and S4201-D1 transcriptomes, respectively. The Q30 percentages (sequencing error rate < 0.1%) of S4201-W and S4201-D1 were 90.20% and 90.43%, respectively. The GC content of the unigenes of S4201-W and S4201-D1 was 51.00% and 49.00%, respectively (Table 1). Afterwards, all of the high-quality reads were assembled into 17,923 unigenes with an average length of 1287.45 bp and an N50 of 1970 bp using the Trinity software. The length distribution of all unigenes is shown in Figure S3. And the GC content frequency distribution is shown in Figure S4. The sequences from both the libraries were deposited at NCBI and can be accessed in the Short Read Archive (SRA) under the accession numbers SRR2352154 and SRR2153022.

### 2.2. Functional Annotation and Classification of Unigenes

Annotation of the unigenes was performed against GenBank non-redundant (NR), SwissProt, Clusters of orthologous groups for eukaryotic complete genomes (KOG), Gene Ontology (GO) and Kyoto Encyclopedia of Genes and Genomes (KEGG) databases using BLASTX search with a cut-off *E* value of 10^−5^. Here, 14,255 unigenes (79.53%) were annotated in these databases (Table 2).

**Figure 1:**
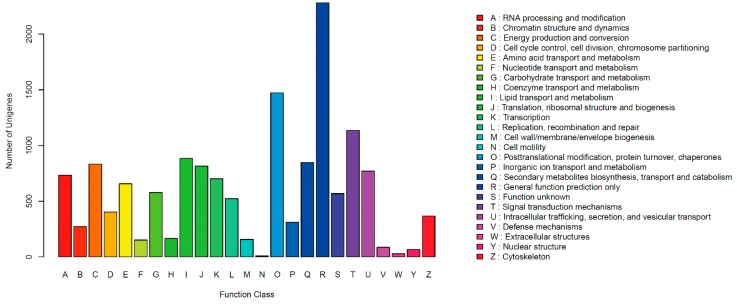
KOG of unigenes of *Shiraia bambusicola*. KOG Functional Classification of transcriptome. In all, 7225 unigenes were clustered into 25 functional categories.

Clusters of orthologous groups for eukaryotic complete genomes (KOG) describesorthologous groups for eukaryotic genomes, and each protein is assumed to be from an ancestor protein. In all, 7225 unigenes were clustered into 25 functional categories (Figure 1). Among the 25 KOG categories, the cluster for ”General function prediction only” (2280, 31.56%) was the largest group, followed by “Posttranslational modification, protein turnover, chaperones” (1472, 20.37%), “Signal transduction mechanisms” (1136, 15.72%), “Lipid transport and metabolism” (883, 12.22%) and “Secondary metabolites biosynthesis, transport and catabolism” (847, 11.72%); only a few unigenes were assigned to the “Extracellular structures” and “Cell motility” groups.

**Figure 2:**
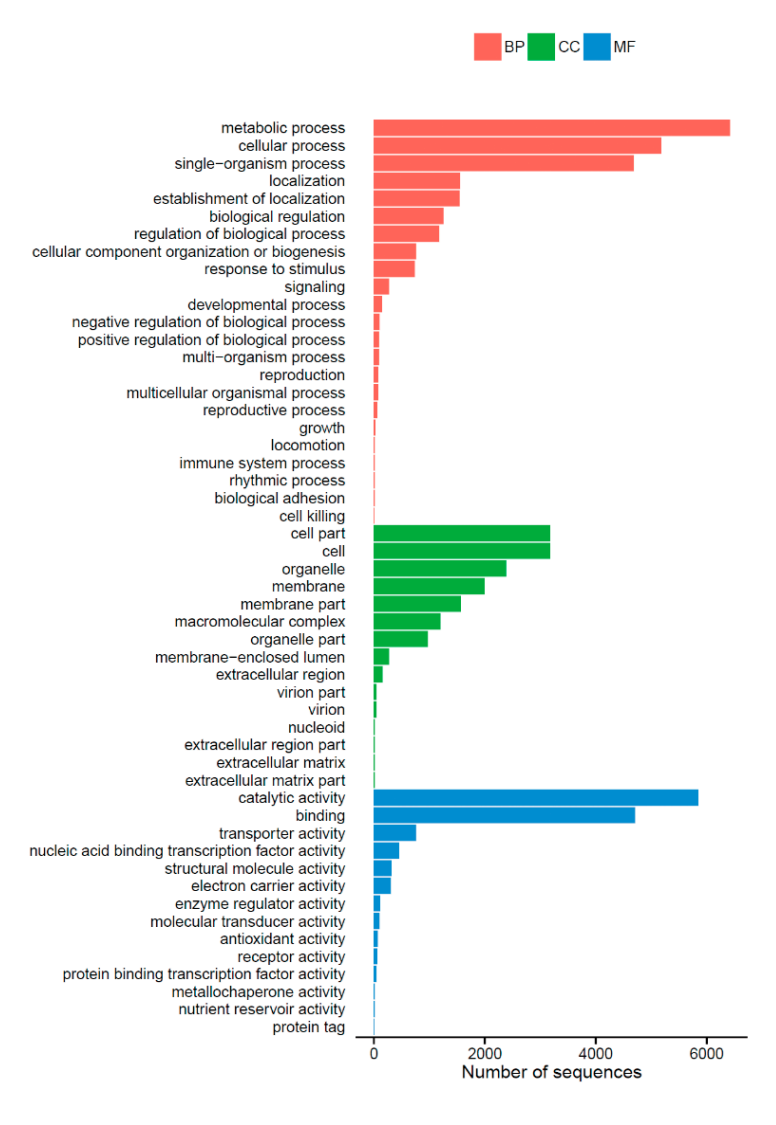
Gene Ontology classification of *Shiraia bambusicola* transcriptome. A total of 9647 (53.82%) unigenes were assigned to their main GO categories (Biological processes (BP), Cellular components (CC), molecular functions (MF)), including 52 sub-categories.

Gene ontology (GO) is an international standardized gene functional classification system that provides a controlled vocabulary for describing the properties of genes and their products. The three main independent categories, including “biological process”, “cellular component” and “molecular function”, are shown in Figure 2. A total of 9647 (53.82%) unigenes were assigned to their main GO categories, including 52 sub-categories. Under the biological process group, the largest proportions were metabolic process (GO: 0008152) and cellular process (GO: 0009987). Within the cellular component, cell (GO: 0005623) and cell part (GO: 0044464) were the largest groups by far. Catalytic activity (GO: 0003824) and binding (GO: 0005488) represented the majority of terms in the molecular function category.

To further identify the active biological pathways, unigenes were searched against pathway collections in the Kyoto Encyclopedia of Genes and Genomes (KEGG) pathway database, which can analyze gene functions systematically and integrate chemical, genomic and systemic functional information [22,23]. A total of 3581 unigenes were annotated and assigned to 323 different pathways (Table S1). “Ribosome” (ko03010, 253 unigenes) was the most enriched pathway, followed by “Biosynthesis of amino acids” (ko01230, 238 unigenes), “Carbon metabolism” (ko01200, 231 unigenes) and “Oxidative phosphorylation” (ko00190, 168 unigenes).

### 2.3. Transcripts Differentially Expressed between the Wild Type and Mutant

Based on the criteria of FDR ≤ 0.05 and |log_2_ratio| ≥ 1, the results show that 904 unigenes were found to be differentially expressed; 716 unigenes were up regulated in S4201-D1, and 188 unigenes were down regulated. We selected 97 significant differentially expressed genes, the detailed lists are given in Table 3. GO-enrichment was performed against all of the DEGs to research the relationship between genetics and phenotype. We then mapped all DEGs to each term of the GO database and calculated the gene numbers from each GO term.

Oxidation-reduction process and transmembrane transport were prominently enriched in biological process. Integral components of the membrane and ribosome were significantly enriched in cellular component. Oxidoreductase activity and heme binding were the majority of terms in molecular function (Table S2). Taken together, these results reveal that during HA production, the main processes are oxidation-reduction and biosynthesis; however, transmembrane transport processes are active when HA needs to be exported.

In the meantime, we identified significantly enriched metabolic pathways by enrichment analysis of the DEGs and mapped 904 unigenes to 201 pathways (Table S3). Among them, the pathways with the most representation by unigenes were “Ribosome” and “Oxidative phosphorylation”.

In the KEGG pathway enrichment analysis, genes of certain pathway in S4201-W showed upregulation compared with S4201-D1, and these pathways included “Glycolysis/Gluconeogenesis” (ko00010), “Fructose and mannose metabolism” (ko00051), “Tyrosine metabolism” (ko00350), “Phenylalanine metabolism” (ko00360), “Inositol phosphate metabolism” (ko00562), “Pyruvate metabolism” (ko00620), “Methane metabolism” (ko00680) and “Carbon metabolism” (ko01200). The upregulated unigenes will likely increase production of the two substrates acetyl-CoA and malonyl CoA, either directly or indirectly, which can provide enough raw material for HA biosynthesis.

The metabolism of S4201-D1, which lacks HA production ability, is more vigorous than S4201-W in fatty acid, amino acid and most saccharide synthesis. This suggests that in S4201-D1, it is more helpful to store nutrition and defend against environmental stresses. There are various possible reasons for this phenomenon: (1) Because the secondary metabolism in S4201-D1, which produced HA, was diminished or eliminated, the primary metabolism that produced carbohydrates, amino acids and lipids was reinforced; (2) HA is cytotoxic and can damage cells and decrease cellular activity. Without HA, the cellular activity of S4201-D1 increased, and the primary metabolism also increased; (3) Without HA, the defense mechanism of S4201-D1 was also diminished or eliminated. This saved materials and energy for other metabolic processes; (4) To repair UV damage, S4201-D1 increased its metabolism of carbohydrates, amino acids and lipids. The possible reasons are based on the speculation that they might be cause and effect relationship, but they may also be correlations. We need further studies to identify whether the relationship between these up regulated genes and HA production is correlation or a cause and effect relationship.

### 2.4. Candidate Genes Involved in HA Biosynthesis and a Putative Biosynthetic Pathway

The genes involved in the biosynthesis and modification of many secondary metabolites in fungi are organized in clusters [24]. We found seven of the 904 differentially expressed genes may be candidate genes involved in HA biosynthesis. These transcripts showed significantly higher expression in S4201-W (with HA production) compared with S4201-D1 (without HA production). They were not expressed or were at very low level in S4201-D1. These seven unigenes encode multicopper oxidase, fasciclin, polyketide synthase, o-methyltransferase/FAD-dependent monooxygenase, o-methyltransferase, hydroxylase and FAD/FMN-dependent oxidoreductase respectively. They were found to have high identity with their corresponding homologous genes through annotation using NR databases. Finally, their sequence information was completely matched with the “*Shiraia* sp. slf14 hypocrellin A biosynthesis gene cluster, complete sequence” (GenBank: KM434884.1, unpublished results) in the NCBI database. Based on the above results, we identified these seven genes as the putative genes of HA biosynthesis.

**Figure 3:**
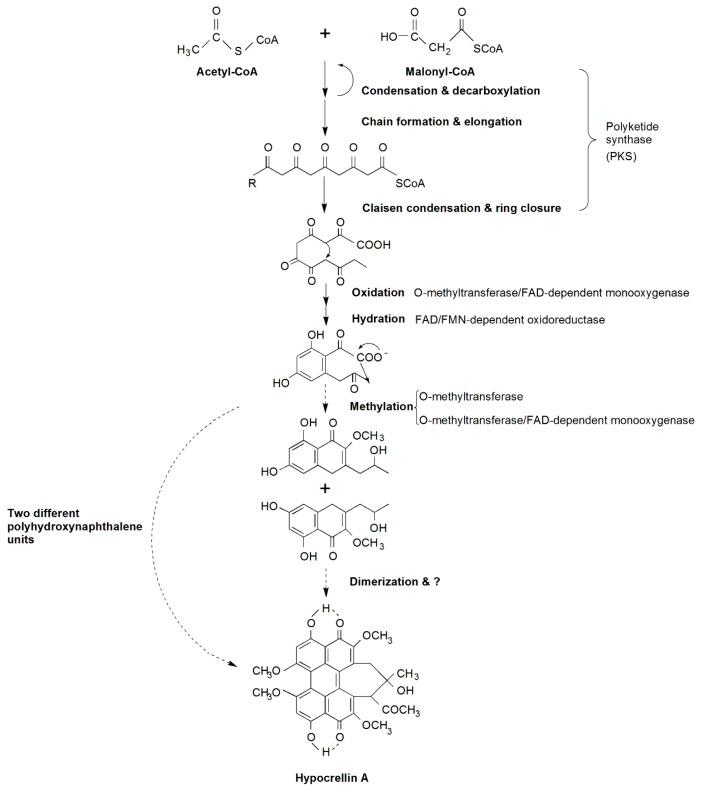
Putative HA biosynthetic pathway-related genes and products in *Shiraia bambusicola.* Reactions catalyzed at every step are shown. The broken arrow represents putative steps, in which the enzymes involved are not yet clear.

We proposed a putative HA biosynthetic pathway, shown in Figure 3. It is based on the model of cercosporin biosynthesis, which shares a similar structure, coding genes and is also a perylenequinoid pigment. Similar to cercosporin biosynthesis [16], acetyl-CoA and malonyl-CoA are the precursor and substrate of HA. The first step of the biosynthesis pathway has been predicted to be condensation and decarboxylation of acetyl-CoA (starter) and malonyl-CoA (extender) by the functional domains of the polyketide synthase. The chain of carbon elongates to form a pentaketide molecule that subsequently undergoes cyclization, then o-methyltransferase/FAD-dependent monooxygenase, FAD/FMN-dependent oxidoreductase and o-methyltransferase catalyze a series of oxidation, hydration and methylation reactions to yield cyclized polyhydroxynaphthalene units. Thus, HA is likely formed by dimerizing two polyhydroxynaphthalene units, and then undergoes other unknown modification reactions. Cercosporin has a bilateral symmetric structure, but HA does not. Hence, it is likely that before dimerization, HA has formed two different polyhydroxynaphthalene units, or the final form of HA is accomplished by dimerization of the two identical polyhydroxynaphthalene units which were similar to cercosporin. Once synthesized, HA must be exported out of cells, presumably by a major facilitator superfamily (MFS) transporter, but our RNA-Seq data showed complete opposite expression patterns that were not concordant with the MFS transporter in *Cercospora* species. In addition, other DEGs are likely to be involved in and regulate the biosynthesis of HA, but this still needs further research. In all, we identified a large number of candidate genes involved in HA biosynthesis by analyzing the RNA-Seq data. To identify whether a gene takes part in the biosynthesis of HA or not and to understand its function, we need to do future studies, such as gene cloning, analysis of its structure, and function verification.

By comparing these gene sequences between the wild type and the mutant, we found that there are a total of three single nucleotides polymorphisms, of which one resulted in amino acid polymorphisms. This gene encodes o-methyltransferase/FAD-dependent monooxygenase and its N-terminal amino acid sequence is similar to that of the transcription enhancer AFLS (formerly AFLJ) involved in aflatoxin biosynthesis. Unfortunately, the mutation site may not be in the functional domains. Therefore, we suggested that the cause of the lack of HA biosynthesis might be in transcriptional regulation, rather than genetic mutations in candidate genes involved in HA biosynthesis. Gene expression in eukaryotes is regulated primarily at the level of transcription and we proposed the following reasons why S4201-D1 cannot produce HA at transcription level: (1) The mutations in the specific transcription factor of the HA biosynthesis gene cluster may lead to that the transcription complexes cannot transcribe normally; (2) Trans-acting factors cannot bind to the mutant *cis*-acting elements and this blocks the initiation of transcription; (3) UV might cause the formation of new silencers which can affect these genes, or cause the formation of binding sites for repressor proteins in the upstream region of the transcription start sites; (4) Moreover, we found that there are no significant differences in expression of transcription factors between the wild type and the mutant, and the cause of the lack of HA biosynthesis may not be the change in numbers of transcription factors. Therefore we still need to study the gene expression mechanism of HA biosynthesis at the transcriptional level.

### 2.5. Autodefense by Shiraia bambusicola against HA

Apart from studying the biosynthesis of HA, autodefense against HA also needs to be understood to improve HA production and *S. bambusicola* self-resistance. The candidate genes that are involved in HA biosynthesis were not expressed, this means S4201-D1 cannot produce HA. Because of the absence of HA, S4201-D1 would not generate reactive oxygen species (ROS), and further related oxidative damages would not happen yet. Meanwhile, the redox reaction in S4201-D1 was not as remarkable as it was in S4201-W, and we will discuss the self-defense mechanism of *S. bambusicola* as follows. In the presence of light and oxygen, photosensitizers can generate ROS, such as singlet oxygen (^1^O_2_) and superoxide radical (O^2−^), which can damage proteins, lipids and nucleic acids [25,26]. Due to its mode of action, HA has almost universal toxicity to cells, and it can damage not only plants but also bacteria, fungi, viruses and tumors. *S. bambusicola*, however, not only synthesizes, but also is resistant to, high concentrations of HA in culture. Cellular resistance mechanisms against perylenequinone photosensitizers such as cercosporin are a basic biological phenomenon. Cercosporin serves as a model system in which to study cellular resistance to ^1^O_2_ in HA. Several ^1^O_2_ quenchers have been characterized in previous research [27]. Carotenoids are one of the most effective quenchers of cellular ^1^O_2_ that exist in biological systems, and they are one of the most effective defenses against the compounds producing ^1^O_2_ [28,29,30]. In our RNA-Seq data, two genes involved in carotenoid biosynthesis were upregulated in S4201-W compared with S4201-D1. And we found that the two genes encoded lycopene beta-cyclase and torulene dioxygenase respectively. Therefore, carotenoid is likely to be involved in autodefense mechanisms of HA. PDX1 and PDX2, two genes that encode enzymes involved in the vitamin B6 biosynthesis pathway, were identified as essential for cercosporin resistance [31,32]. After this discovery, vitamin B6 was found to quench ^1^O_2_ superoxide effectively and to have antioxidant activity [33,34]. *Cercospora nicotianae* mutants, which do not have a functional vitamin B6 biosynthetic gene, lose their cellular resistance to cercosporin [31]. Our RNA-Seq data showed that the gene, in *S. bambusicola* S4201-W, encoding pyridoxine 4-dehydrogenase involved in vitamin B6 biosynthesis was expressed at higher levels than in mutant S4201-D1. Therefore, VB6 is likely to be involved in the autodefense mechanism of HA. Many photosensitizers are converted to a non-phototoxic form when reduced, and colorless forms lack normal visible light absorption. Cercosporin in these reduced derivatives absorb about half the amount of light compared with cercosporin [35]. The cercosporin in the fungal cell is reduced to a nontoxic mode. When the cercosporin has been released, it reoxidizes to its photoactive form spontaneously [36]. Therefore, reduced cercosporin is used as a cellular resistance mechanism in *Cercospora* species. Meanwhile, our DEG data mainly found enrichment in oxidation-reduction processes within the biological process group. In addition, dioxin degradation (ko00621), polycyclic aromatic hydrocarbon degradation (ko00624), naphthalene degradation (ko00626) and degradation of aromatic compounds (ko01220) were significantly enriched pathways, in which certain unigenes were upregulated in S4201-W compared with S4201-D. These pathways refer to strong reduction processes that can decrease cell damage from an oxidizing agent. These results are in accordance with biological characteristics of HA.

### 2.6. Real-Time PCR Analysis of Several Transcripts

**Figure 4:**
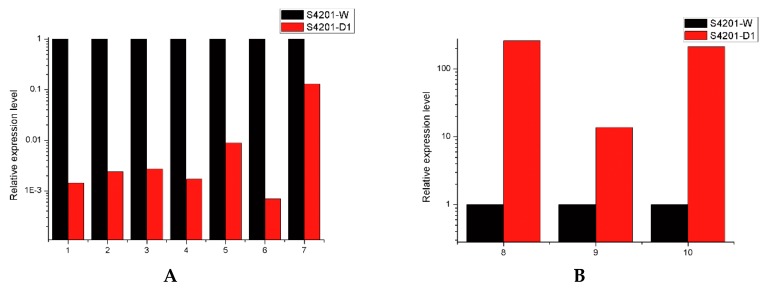
Quantitative RT-PCR validation. The relative expression levels of mRNA were normalized with internal reference gene (GAPDH) and relative expression to the corresponding values of S4201-W (control) were given an arbitrary value of 1. (**A**) 1: Multicopper oxidase, 2: Fasciclin, 3: Polyketide synthase, 4: O-methyltransferase/FAD-dependent monooxygenase, 5: Hydroxylase, 6: FAD/FMN-dependent oxidoreductase, 7: Pyridoxal reductase; (**B**) 8: Aldehyde dehydrogenase (NAD^+^), 9: Cytochrome P450, 10: Alcohol dehydrogenase.

We selected six unigenes involved in HA biosynthesis and four DEGs randomly chosen through quantitative real time PCR (qRT-PCR) to validate the changes in gene expression identified by RNA-Seq. The results showed that the unigenes that encode multicopper oxidase, fasciclin, polyketide synthase, o-methyltransferase/FAD-dependent monooxygenase, hydroxylase FAD/FMN-dependent oxidoreductase and pyridoxal reductase, were significantly higher in S4201-W, whereas aldehyde dehydrogenase (NAD^+^), cytochrome P450, alcohol dehydrogenase encoding genes were mainly expressed in S4201-D (Figure 4). The expression levels of these 10 samples were highly consistent with the RNA-Seq data, suggesting that the RNA-Seq data are reliable.

## 3. Experimental Section

### 3.1. Materials

The wild strain S4201-W was isolated from the fruiting bodies of the *Shiraia* *bambusicola* P. Henn collected in Anji in the Zhejiang Province of China, and screened as a hypocrellin-producing strain. No specific permissions were required for the described field studies. The location is not privately-owned or protected, and the field studies did not involve endangered or protected species. After molecular identification based on nuclear ribosomal internal transcribed spacer (rDNA ITS, the ITS region is the most frequently sequenced genetic marker of fungi), the strain was preserved in the Microbial Culture Center of College of Life Sciences, Nanjing Normal University, China. The strain S4201-W was cultured on potato dextrose agar (PDA) and washed with sterile water to prepare the spore suspension, and the concentration was adjusted to 1 × 10^6^ spores/mL. The spore suspension was irradiated by a 800 mj/cm^2^ dose of ultraviolet (UV) radiation treatment with a UV-light cross-linker (UV light source). We selected the sample that did not produce HA on PDA. HA was measured by high-performance liquid chromatography (HPLC) with HA standard reagent. *S. bambusicola* S4201-W has excellent HA production, while the mutant S4201-D1 does not. Then, S4201-W and S4201-D1 were inoculated into potato dextrose broth (PDB) medium with Triton X-100 and cultured in the dark at 28 °C on a shaker at 150 rpm/min for 82 h [37]. Fermentation liquid was centrifuged at 3000 rpm for 3 min at room temperature in a microcentrifuge, and the supernatant was removed. Mycelia of each sample were immediately frozen and stored at −80 °C.

### 3.2. RNA Isolation and Illumina Sequencing

The samples were collected from 82-h-old mycelia, respectively. Total RNA from each sample was extracted using the mirVana™ miRNA Isolation Kit without phenol (Ambion, Inc., Austin, TX, USA) and treated with RNase free DNaseI according to manufacturer’s instructions. The quality of RNA was confirmed by spectrophotometer (NanoDrop ND-1000, NanoDrop Technologies, Wilmington, DE, USA) and agarose gel electrophoresis before further processing. Poly (A) mRNA from total RNA was enriched by oligo (dT) magnetic beads and then broken into short fragments to synthesize the first strand of cDNA by random hexamer-primer. DNA polymerase I and RNaseH were used to synthesize the second strand of cDNA and repair the ends, which were connected using sequencing adapters. Afterwards, cDNA libraries were constructed by PCR, and the sequences were analyzed using the Illumina HiSeq^TM^ 2500 sequencing platform (commercial service) at Shanghai OE Biotech Co., Ltd. (Shanghai, China).

### 3.3. De Novo Transcriptome Assembly and Annotation

The raw reads were cleaned by removing adaptor sequences, empty reads and low-quality reads. In our research, the Trinity (vesion: trinityrnaseq_r20131110) program was used to assemble high quality reads for each sample [38]. Because *S. bambusicola* has no reference genome in public databases, reads needed to be assembled *de novo*. Based on a BLASTX search (*E*-value < 10^−5^), the functional annotation of these unigenes were performed using different protein databases, including the NCBI NR, Kyoto Encyclopedia of Genes and Genomes (KEGG), SwissProt and KOG databases. GO annotations based on the BLAST2GO program were used to further analyze the functional classifications of these unique sequences [39].

### 3.4. Analysis of Differential Gene Expression

The unigene expression was determined by using the reads per million kilobases per million mapped reads (RPKM) method to normalize the read counts between the samples [40,41]. Differentially expressed genes (DEGs) were identified with a false discovery rate (FDR) ≤ 0.05 and |log_2_ratio| ≥ 1. To find out what was significantly differentially expressed between S4201-W and S4201-D1, the *p*-values ≤ 0.05 and fold changes ≥ 2 were used as the estimate threshold.

### 3.5. Quantitative Real-Time PCR Analysis

Quantitative reverse-transcriptase polymerase chain reaction (qRT-PCR) of differentially expressed genes in S4201-W and S4201-D1 was used to evaluate the quality of the sequence assembly. The expression patterns of ten transcripts were monitored, with three independent replicates of each transcript. Glyceraldehyde-3-phosphate dehydrogenase (GAPDH) was used as an internal reference gene. RNA from each sample was isolated using the mirVana™ miRNA Isolation Kit without phenol and treated with RNase-free DNaseI according to the manufacturer’s instructions. The first strand of cDNA was synthesized by the use of PrimeScript^TM^ RT Master Mix (TaKaRa, Dalian, China). qRT-PCR was performed in an StepOnePlus^TM^ Real-Time PCR System (Applied Biosystems, USA) using a SYBR Premix Ex Taq^TM^ II (TaKaRa, Dalian, China) and the conditions as follows: 95 °C for 30 s followed by 40 cycles of 95 °C for 5 s, 60 °C for 30 s. A melting curve was generated to test specificity of the products produced by the qRT-PCR. The results were calculated using the 2^−ΔΔ*C*t^ method [42]. Primer sequences are presented in the supporting information (Table S4).

## 4. Conclusions

Hypocrellin compounds are perylenequinonoid pigments with excellent antiviral and antitumor properties. However, their complex metabolic pathway remains unknown. Our study is the first attempt to use the Illumina/Solexa deep sequencing platform for *de novo* sequencing and assembly of *S. bambusicola* without a reference genome. A large number of transcripts differentially expressed between the wild strain S4201-W and mutant S4201-D1 have been identified, especially the ones involved in the biosynthesis of HA. In addition, we proposed a putative HA biosynthetic pathway. This study facilitates understanding of the gene expression and functional genomics of *S. bambusicola* and provides a valuable set of public information that can be used to further explore the molecular mechanisms of various metabolic pathways. The transcriptome sequence data will allow for increased HA production using biotechnological applications and metabolic engineering in the near future.

## Figures and Tables

**Table 1:** Statistics of transcriptome sequencing.

Sample	S4201-W	S4201-D1
Raw reads	38,230,680	39,301,794
Raw bases	4,778,835,000	4,912,724,250
Clean reads	38,056,034	39,086,896
Clean bases	4,751,476,731	4,880,073,139
Valid ratio (base)/%	99.42	99.33
Q30/%	90.20	90.43
GC content/%	51.00	49.00

**Table 2:** Summary of annotations of unigenes in public databases.

Database	No. of Matched Unigenes	Percentage/%
Nr	14,243	79.47
SWISS-PROT	9011	50.28
KOG	7225	40.31
KEGG	3581	19.98
GO	9647	53.82

**Table 3:** Significant differentially expressed genes between S4201-W and S4201-D1.

Gene ID	Description	|Log_2_Fold Change|	*p*-Value	Up_Down *
comp12495_c0_seq3	o-methyltransferase/FAD-dependent monooxygenase	19.98004	0.0000384	down
comp12440_c0_seq1	hydroxylase	17.92012	0.0000204	down
comp12566_c0_seq1	fasciclin	17.23691	0.000032	down
comp12222_c0_seq4	o-methyltransferase/FAD-dependent monooxygenase	16.88793	0.0000458	down
comp11902_c0_seq9	polyketide synthase	15.5618	0.000334	down
comp9636_c0_seq2	similar to NADPH-cytochrome P450 reductase	15.35454	0.000492	down
comp11380_c0_seq2	putative multicopper oxidase	14.86465	0.001313	down
comp6304_c0_seq2	carboxypeptidase	14.7857	0.00154	down
comp11297_c0_seq3	putative MFS drug efflux transporter protein	14.73989	0.00169	down
comp11445_c0_seq2	amino acid transporter	13.91772	0.008498	down
comp10001_c0_seq5	FAD/FMN-dependent oxidoreductase	13.86749	0.009314	down
comp17278_c0_seq1	plastin-3	13.68616	0.012799	down
comp11463_c0_seq1	glycoside hydrolase family 95 protein	13.57229	0.015462	down
comp17195_c0_seq1	mannitol 1-phosphate dehydrogenase 1	13.24513	0.02557	down
comp15202_c0_seq1	alpha/beta-hydrolase	12.89003	0.040099	down
comp17704_c0_seq1	chitin synthase D	12.7117	0.04854	down
comp11073_c0_seq3	ATP synthase subunit 6	20.3094	0.0000172	up
comp15750_c0_seq1	cytochrome oxidase subunits 1 and 2 polyprotein	18.0501	0.00000343	up
comp6469_c0_seq2	putative methyltransferase type 11 protein	17.7353	0.00000373	up
comp2455_c0_seq1	similar to MFS peptide transporter Ptr2	17.5382	0.00000412	up
comp10718_c0_seq3	heat shock protein 90a	17.2418	0.00000508	up
comp17877_c0_seq1	NADH dehydrogenase subunit 1	16.9127	0.00000697	up
comp7051_c0_seq1	putative family transcriptional regulator protein	16.8352	0.00000761	up
comp2192_c0_seq2	decaprenyl-diphosphate synthase subunit 1	16.4804	0.000012	up
comp7011_c0_seq2	60S ribosomal protein L2	16.4285	0.0000129	up
comp7761_c0_seq1	similar to kelch repeat protein	16.2001	0.0000182	up
comp7729_c0_seq1	similar to protein phosphatase 2C	15.9167	0.0000292	up
comp22695_c0_seq1	similar to sugar transporter	15.5938	0.0000525	up
comp16042_c0_seq1	elongation factor 3	15.5433	0.0000578	up
comp15117_c0_seq1	chitin synthase	15.5174	0.0000609	up
comp5090_c0_seq1	phosphate permease (PHO89/Pi cotransporter PHO89)	15.3805	0.0000805	up
comp1206_c0_seq1	laccase	15.3337	0.0000889	up
comp7656_c0_seq1	chitin synthase 4	15.2418	0.000108	up
comp9653_c1_seq2	glutathione *S*-transferase GliG-like, putative	15.1638	0.000129	up
comp8271_c0_seq2	efflux pump antibiotic resistance protein	15.0455	0.000168	up
comp10576_c0_seq1	similar to 40s ribosomal protein S15	15.0162	0.00018	up
comp18277_c0_seq1	similar to 60s acidic ribosomal protein P0	15.0088	0.000183	up
comp7104_c0_seq1	high affinity glucose transporter	14.9635	0.000203	up
comp9911_c0_seq2	cytochrome P450 monooxygenase, putative	14.7665	0.000318	up
comp15511_c0_seq1	glycoside hydrolase family 105 protein	14.7576	0.000324	up
comp5983_c0_seq2	aspartyl-tRNA synthetase	14.7218	0.000352	up
comp9603_c0_seq2	MFS general substrate transporter	14.6184	0.000445	up
comp12046_c0_seq3	chp3	14.6086	0.000456	up
comp1492_c0_seq1	putative duf341 domain protein	14.5174	0.000561	up
comp9605_c0_seq1	similar to WSC domain-containing protein	14.4313	0.000683	up
comp8564_c0_seq1	conserved hypothetical protein	14.2793	0.000966	up
comp17817_c0_seq1	cysteine desulfurase, mitochondrial precursor	14.2163	0.001115	up
comp20949_c0_seq1	similar to beta-1,3-glucan synthase	14.1368	0.001334	up
comp6798_c0_seq1	similar to 40S ribosomal protein S9	14.1368	0.001334	up
comp16828_c0_seq1	transcription factor PacC	14.1093	0.001419	up
comp8237_c0_seq2	similar to delta-12 fatty acid desaturase	14.1093	0.001419	up
comp9104_c0_seq1	eukaryotic translation initiation factor 1A	14.0813	0.001511	up
comp9278_c0_seq1	transport protein, putative	14.0813	0.001511	up
comp5653_c0_seq1	40S ribosomal protein S5	14.0671	0.00156	up
comp8053_c0_seq1	putative short-chain dehydrogenase reductase family protein	14.0383	0.001663	up
comp2722_c0_seq1	integral membrane protein, putative	14.0088	0.001774	up
comp8290_c0_seq2	ferric reductase transmembrane component	13.9939	0.001834	up
comp6711_c0_seq1	conserved hypothetical protein	13.9788	0.001895	up
comp2304_c0_seq1	cytochrome P450	13.8846	0.002321	up
comp2698_c0_seq1	short chain dehydrogenase family protein	13.8846	0.002321	up
comp18073_c0_seq1	mitochondrial fusion GTPase protein	13.8683	0.002403	up
comp15182_c0_seq1	vacuolar protein sorting-associated protein 45	13.8518	0.002488	up
comp9144_c0_seq2	cytochrome P450 monooxygenase	13.8518	0.002488	up
comp82_c0_seq1	60S ribosomal protein L14	13.8183	0.002668	up
comp20237_c0_seq1	similar to aromatic amino acid aminotransferase	13.6757	0.003561	up
comp7159_c0_seq1	similar to 40S ribosomal protein S14	13.6377	0.003835	up
comp7442_c0_seq1	actin	13.5174	0.004829	up
comp542_c0_seq1	pyrrolocin enoylreductase	13.475	0.005228	up
comp2644_c0_seq1	heat shock protein Hsp88	13.4089	0.005901	up
comp2635_c0_seq1	C protein immunoglobulin-A-binding beta antigen	13.3862	0.006148	up
comp8431_c0_seq2	mitochondrial citrate synthase	13.3862	0.006148	up
comp20213_c0_seq1	similar to elongation of fatty acids protein	13.3631	0.006406	up
comp949_c0_seq1	similar to adenosine deaminase family protein	13.2163	0.00823	up
comp2229_c0_seq2	similar to zinc/cadmium resistance protein	13.1368	0.009341	up
comp6853_c0_seq2	cyclin	12.9325	0.012519	up
comp15194_c0_seq1	RNA polymerase II mediator complex component	12.8352	0.014188	up
comp617_c0_seq1	chloroperoxidase	12.8012	0.014797	up
comp15885_c0_seq1	similar to heat shock protein	12.7665	0.015435	up
comp8520_c0_seq1	Beta-lactamase family protein	12.7665	0.015435	up
comp14744_c0_seq1	SH3 domain containing protein	12.7308	0.016106	up
comp18002_c0_seq1	NADH-ubiquinone oxidoreductase	12.6943	0.016811	up
comp2251_c0_seq1	similar to cystathionine beta-synthase	12.6943	0.016811	up
comp14820_c0_seq1	geranylgeranyl diphosphate synthase, putative	12.6184	0.018339	up
comp16310_c0_seq1	conserved hypothetical protein	12.6184	0.018339	up
comp3019_c0_seq1	similar to benzoate 4-monooxygenase cytochrome P450	12.4533	0.021976	up
comp17256_c0_seq1	similar to phosphoglucomutase	12.3631	0.024168	up
comp8045_c0_seq3	MFS transporter	12.2163	0.028088	up
comp14626_c0_seq1	similar to 26 proteasome complex subunit Sem1	12.1093	0.031252	up
comp11395_c0_seq4	NAD(P)-binding Rossmann-fold containing protein	12.0528	0.033043	up
comp5357_c0_seq1	TPA: cytochrome P450, putative (Eurofung)	12.0528	0.033043	up
comp16027_c0_seq1	putative cytochrome P450	11.9939	0.034993	up
comp19911_c0_seq1	AF360398_16-methylsalicylic acid synthase	11.9325	0.037123	up
comp5843_c0_seq1	proteasome regulatory subunit 12	11.9325	0.037123	up
comp16424_c0_seq1	microbial terpene synthase-like protein	11.8683	0.03946	up
comp9499_c0_seq2	annexin A7	11.8012	0.042037	up
comp5096_c1_seq1	pyrrolocin synthetase	11.6568	0.048082	up
comp22148_c0_seq1	integral membrane protein	11.6568	0.048082	up

* Significant upregulation or downregulation of genes in S4201-D1 as compared to those in S4201-W.

## Supplementary Materials

Supplementary materials can be found at http://www.mdpi.com/1422-0067/17/3/311/s1.
