# Supplementary material for: De Novo Transcriptome Assembly in Shiraia bambusicola to Investigate Putative Genes Involved in the Biosynthesis of Hypocrellin A
Source: Int J Mol Sci. 2016 Feb 27;17(3):311. doi: 10.3390/ijms17030311 (PMC4813174; doi:10.3390/ijms17030311)
Supplement: Supplementary file 1 [file ijms-17-00311-s001.zip › ijms-113860-Supplementary Materials/ijms-113860-supple.pdf]

Supplementary Materials: *De Novo* Transcriptome Assembly in *Shiraia Bambusicola* to Investigate Putative Genes Involved in the Biosynthesis of Hypocrellin A

Ning Zhao, Xi Lin, Shan-Shan Qi, Zhi-Mei Luo, Shuang-Lin Chen and Shu-Zhen Yan

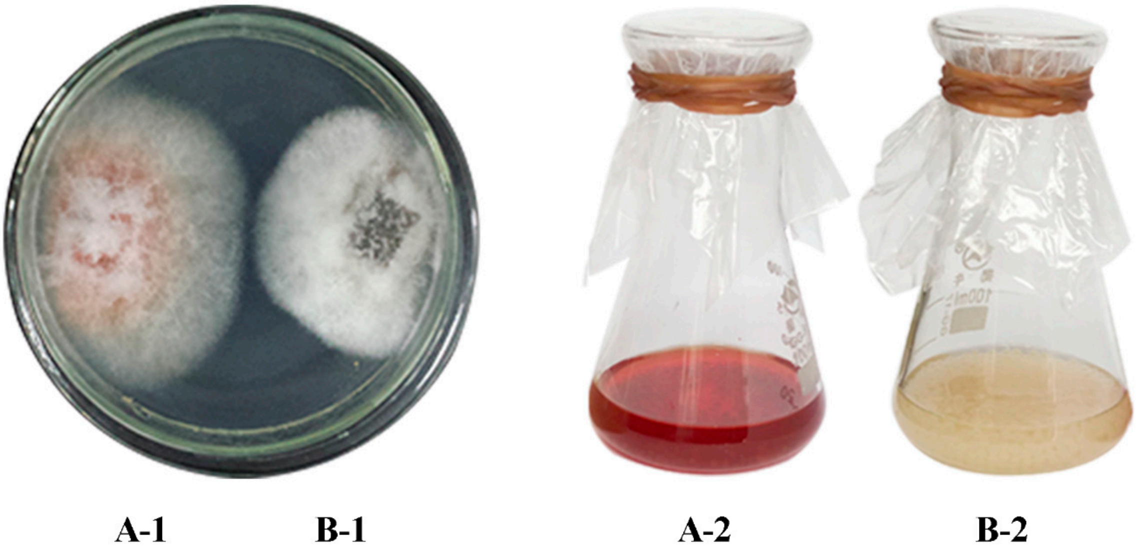

Figure S1. The *S. bambusicola* strains were cultured on PDA and PDB. The wild type S4201-W (**A**) which can produce HA and the mutant S4201-D1 (**B**) which cannot produce HA.

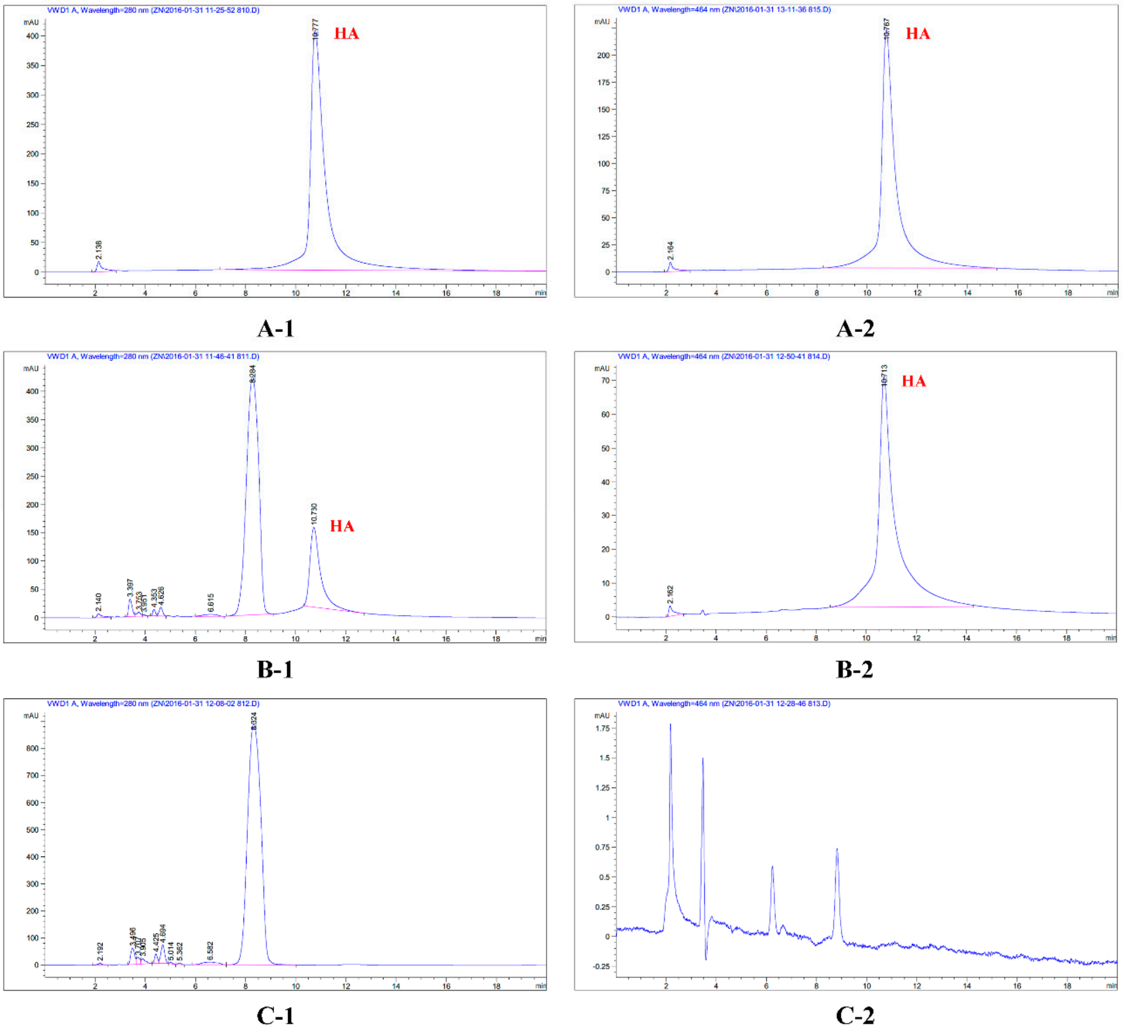

Figure S2. The figures of HA by HPLC. (**A**) HA standard; (**B**) pigments produced by S4201-W; (**C**) pigments produced by S4201-D1 (**A1**, **B1**, **C1**: The absorption peaks at 280 nm; **A2**, **B2**, **C2**: The absorption peaks at 464 nm (maximum absorption wavelength of HA)).

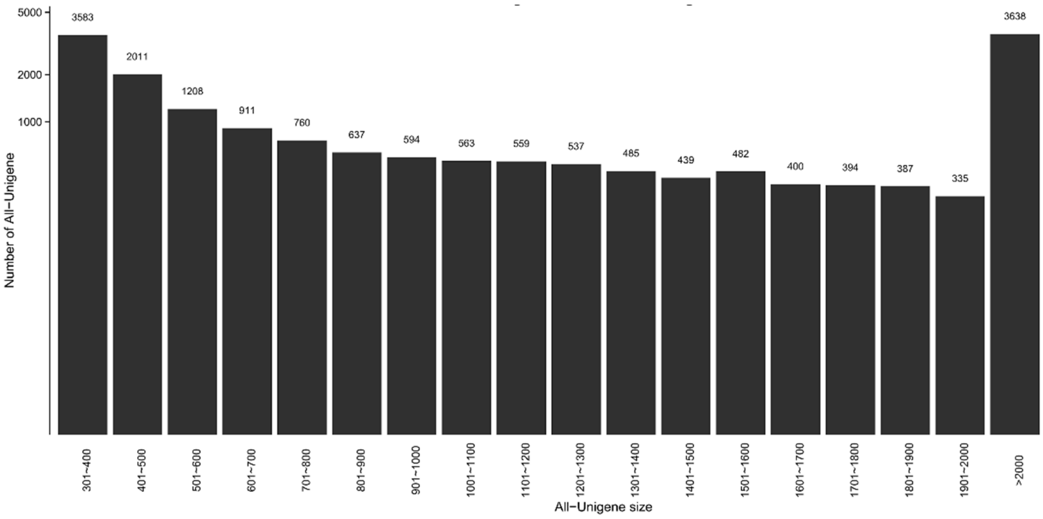

Figure S3. Length distribution of all unigenes.

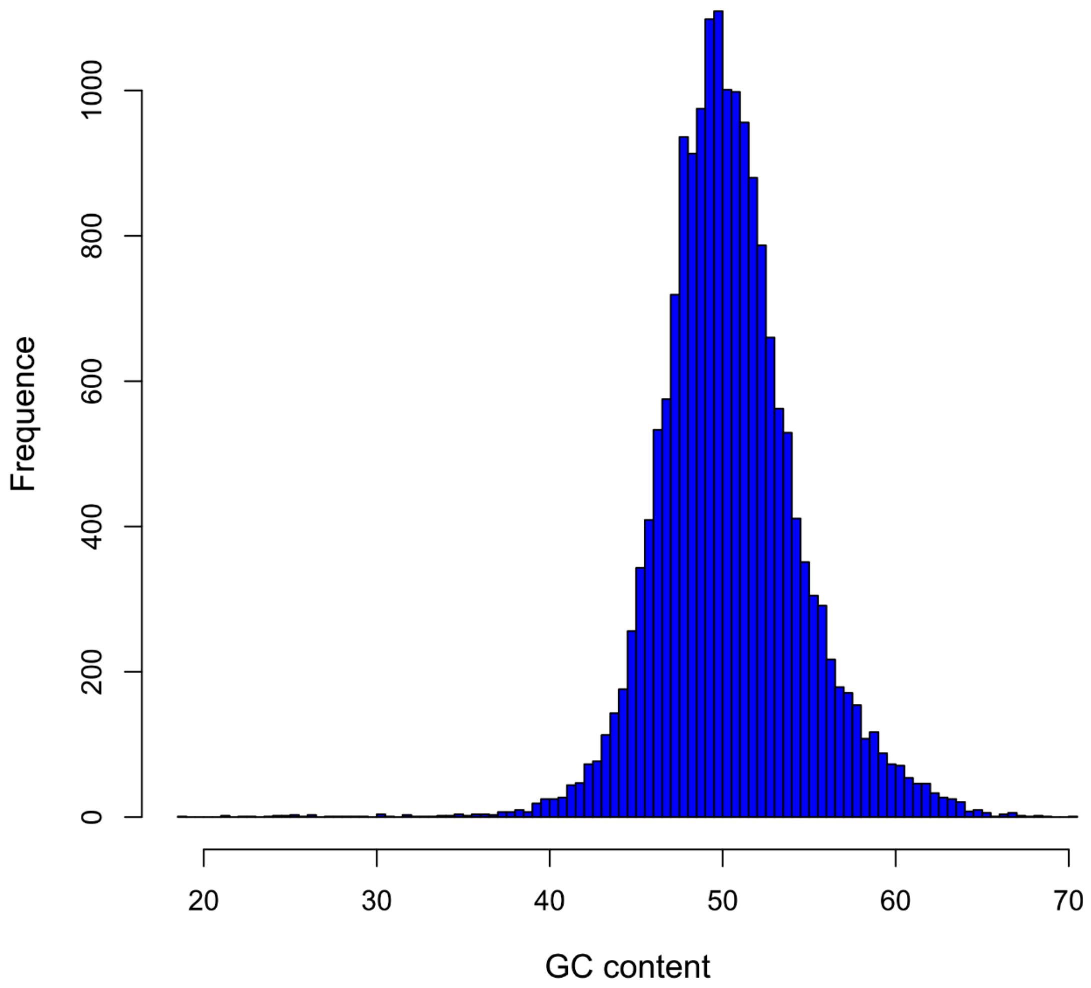

Figure S4. GC content frequence distribution.
